# Assessment, life circumstances, curriculum and skills: Barriers and enablers to student mental wellbeing in distance learning

**DOI:** 10.3389/fpsyg.2023.1076985

**Published:** 2023-02-06

**Authors:** Kate Lister, Kyle Andrews, Jo Buxton, Chris Douce, Jane Seale

**Affiliations:** ^1^Faculty of Learning and Teaching, Arden University, Coventry, United Kingdom; ^2^Faculty of Wellbeing, Education and Language Studies, The Open University, Milton Keynes, United Kingdom; ^3^Institute of Educational Technology, The Open University, Milton Keynes, United Kingdom; ^4^Faculty of STEM, The Open University, Milton Keynes, United Kingdom

**Keywords:** mental health, mental wellbeing, students, compassion, participatory, social model

## Abstract

Student mental wellbeing is increasingly a priority for universities, and this is particularly critical in a distance learning context. Studies have found that studying, academic pressure, university culture and systems can affect students’ mental health. There are increasing calls for universities to take a compassionate, holistic approach to supporting student wellbeing, and identify the barriers that are created by university cultures, systems, pedagogies, curricula, tuition and assessment practices. This study aimed to identify barriers and enablers to student mental wellbeing in distance learning, and students’ recommendations for changes to be made. Using a student survey (N = 584), we identified that assessment and life circumstances were the most significant barriers, while the greatest enablers were building study skills, the people in students’ lives, and curriculum and module content. The study revealed significant demographic differences in how students experience barriers and enablers, and how likely they feel they are to benefit from solutions. Students with disclosed mental health difficulties were consistently more likely to experience barriers than students without a disclosure, while enablers were experienced by all demographic groups. The study concludes that assessment should be prioritised as an area for action.

## 1. Introduction

Student mental health and wellbeing is an increasingly high priority for universities (Evans et al., 2018; Hughes and Spanner, 2019). In the United States, it is estimated that a third of students experience mental health difficulties (Lipson et al., 2018), and in Australia, studies have found consistently higher levels of psychological distress, depression and anxiety in students than in the general public (Stallman, 2010; Larcombe et al., 2016). In the United Kingdom, frequent media attention (e.g., Murugesu, 2019; Weale, 2020) and high profile individual cases (BBC News, 2020) have ensured that student wellbeing is of high priority in sector policy and strategy (Hughes and Spanner, 2019; Universities UK, 2020) and is increasingly high on academic research agendas (e.g., Hartrey et al., 2017; Jones et al., 2018; Ribeiro et al., 2018).

This paper reports findings from a study into barriers and enablers to mental health and wellbeing that distance learning students experience in higher education (HE). Distance learning students are often overlooked in literature relating to student wellbeing; this paper shares insight into their experiences, aiming to answer three research questions:

1. What barriers to mental wellbeing do distance learning students experience, and are particular demographic groups more likely to experience these barriers?
2. What enablers to mental wellbeing do distance learning students experience, and are particular demographic groups more likely to experience these enablers?
3. What changes do distance learning students recommend that would enhance mental wellbeing in distance learning?

Terminology relating to mental health is contentious (Davies, 2013; Hughes and Spanner, 2019). In this paper, we follow the approach adopted by UK HE sector bodies; we use the term ‘mental health’ to signify issues that have been medically diagnosed and ‘mental wellbeing’ to cover a broader spectrum of undiagnosed issues such as anxiety and depression. We define ‘barriers’ and ‘enablers’ as determinants within students’ higher education experiences that have a significant positive or negative impact on their overall mental wellbeing.

## 2. Background and literature

### 2.1. Mental wellbeing in higher education

Research shows that mental health can have a significant impact on students’ likelihood of success, in terms of their likelihood to complete their studies, their academic attainment and their likelihood to progress (Richardson, 2015; Office for Students, 2019; Lister et al., 2021). Studies suggest that higher education (HE) may have a negative effect on students’ mental health. In the United Kingdom, university students’ mental health is consistently found to be lower than the mental wellbeing of the general population of comparative age (Neves and Hillman, 2019; Office of National Statistics, 2020). Studies have found that studying, academic pressure, university culture and systems may be affecting students’ mental health (Tinklin et al., 2005; Brown, 2016; Ribeiro et al., 2018; Winzer et al., 2018; Lee and Kim, 2019); for example, Tinklin et al. found that higher education ‘systems’ and ‘structural issues’, ‘had exacerbated and even created some of the students’ difficulties’ (Tinklin et al., 2005, p: 510). A dataset analysis of 80,509 students attending college counselling centres in the United States, United Kingdom and Canada confirmed this, finding that ‘academic distress’, including ‘academic performance, pressure to succeed, and postgraduation plans’, was the most unique predictor of anxiety (Jones et al., 2018, p: 253).

Ribeiro et al. found in a systematic review that ‘psychological suffering is inherent in academic life’ (Ribeiro et al., 2018, p: 6). And while a certain level of stress is expected as part of academia, there is a strong case that higher education needs to become more compassionate, and should adapt or update some of the systems, structures and academic practices that cause undue mental health difficulties. Students have called for changes to different areas of academia in order to improve mental wellbeing; these include: ‘Academic teachers and teaching practices; student services and support; environment, culture and communication; course design; program administration; assessment; and student society activities’ (Baik et al., 2019, p: 674).

Assessment is particularly identified in the literature as a potential barrier to wellbeing (Jones et al., 2020). Assessment is heavily value-laden, and practice has been slow to evolve; particularly in summative assessment (Boud and Falchikov, 2007; Hanesworth et al., 2019). Galante et al. talk about levels of ‘psychological distress’ during exams (2018), and Jones et al. identify assessment design, collaborative work, challenges of assessment workload and post-assessment feedback as ‘psychological threats’, both in summative and formative assessment (2020). Baik et al. also found that assessment design impacted on wellbeing, with student perceptions of clarity and fairness in design being particularly critical (2019), while Hill et al. highlight impacts of assessment feedback on student wellbeing (2021). Specific assessment activities, such as groupwork, can be a barrier for wellbeing (McPherson et al., 2019), while impacts of power dynamics involved in faculty-centred as opposed to student-centred pedagogies have been found to affect students’ confidence and wellbeing (Felton and Stickley, 2004; Hill et al., 2019). Feeling ‘overwhelmed’ has been linked to student withdrawal (Weller et al., 2018, p: 43), and, of course, failure and fear of failure are also major contributors to student academic stress or distress (Whittle et al., 2020).

Pedagogy and curriculum are also recognised to contain barriers to wellbeing. For example, Tinklin *et al* identified ‘Lack of understanding among lecturers’ and ‘badly designed learning experiences’ as barriers (2005, p: 510), and Baik *et al* found that lack of clarity in teaching materials, low levels of classroom interaction and lack of variety in activities impacted negatively on wellbeing (2019). Specific activities, such as groupwork, can be a barrier for wellbeing (McPherson et al., 2019), while impacts of power dynamics involved in faculty-centred as opposed to student-centred pedagogies have been found to affect students’ confidence and wellbeing (Felton and Stickley, 2004; Hill et al., 2019). Feeling ‘overwhelmed’ by curriculum content has been linked to student withdrawal (Weller et al., 2018, p: 43), and distressing curriculum content has been shown to present particular mental health challenges for some students (Slavin et al., 2014; Bentley, 2017).

Barriers to wellbeing may also be linked with students’ skills and resilience (Houston et al., 2017; Galante et al., 2018; Holdsworth et al., 2018; McAllister et al., 2018). For example, Hewitt and Stubbs identify that difficulties with interpersonal skills, the skills involved in managing workload, and the discipline-specific study skills necessary to achieve good grades, may be a cause of depression, anxiety and stress for students (2017). Similarly, Barrable *et al* found that stress associated with ‘study skills difficulties’, particularly around ‘time management, staying motivated, and memory techniques’ (2018) were a trigger for mental ill health and negative feelings. Galante *et al* posit that lack of resilience in dealing with exam stress causes increases in numbers of students seeking counselling support (2018), and Holdsworth *et al* maintain that students should be taught to develop resilience in higher education in order to deal with ‘constant change and stress’ without negatively affecting their mental health (2018).

In line with broader societal shifts in thinking around mental health (Davies, 2013), there are increasing calls for universities to take a more compassionate, proactive and holistic approach to supporting student wellbeing (Houghton and Anderson, 2017; Hughes and Spanner, 2019; Universities UK, 2020). However, there is a lack of consensus in HE around how best to do this (Hartrey et al., 2017). This has led to a plethora of studies trialling interventions-based approaches such as mindfulness (Galante et al., 2018) or therapy (Viskovich and Pakenham, 2018). These studies generally show only limited or short-term success (Winzer et al., 2018), and have not addressed the underlying issues in university norms and culture. There is a need to take a more social model approach (Oliver, 1983), working in partnership with students (Piper and Emmanuel, 2019; Lister, 2022) and adopting a lens of compassion (Gilbert, 2016), and address the barriers to mental wellbeing within the HE environment, instead of a deficit model focusing only on individuals.

### 2.2. Mental wellbeing in distance education

The need to address barriers to mental wellbeing applies particularly to distance learning. While literature suggests that part-time adult learning can be beneficial for wellbeing (Field, 2009; Waller et al., 2018), evidence suggests that students in distance learning are more likely to disclose an existing mental health difficulty, may be more likely to need support (Barr, 2014) and that their needs and challenges may be less visible to the university (Coughlan et al., 2021; Coughlan and Lister, 2022). For example, in 2018–19, 9.6% of Open University (OU) students (12,813 in total) disclosed a mental health condition compared to the UK HE average of 2.5% (Advance, 2018). Furthermore, the OU’s Access and Participation Plan identifies a consistent module completion gap since 2013, with the overall percentage of students completing modules around 16 percentage points lower for students with mental health disclosures (The Open University, 2019). This has not been sufficiently addressed in the literature; most studies trialling interventions have focused on a campus environment, and many of the solutions posited translate poorly to a distance learning environment. Studies are needed that apply a critical lens to the cultures, systems, pedagogies, curricula, tuition and assessment practices in distance learning, and identify the barriers these raise for students’ mental health.

**Figure 1:**
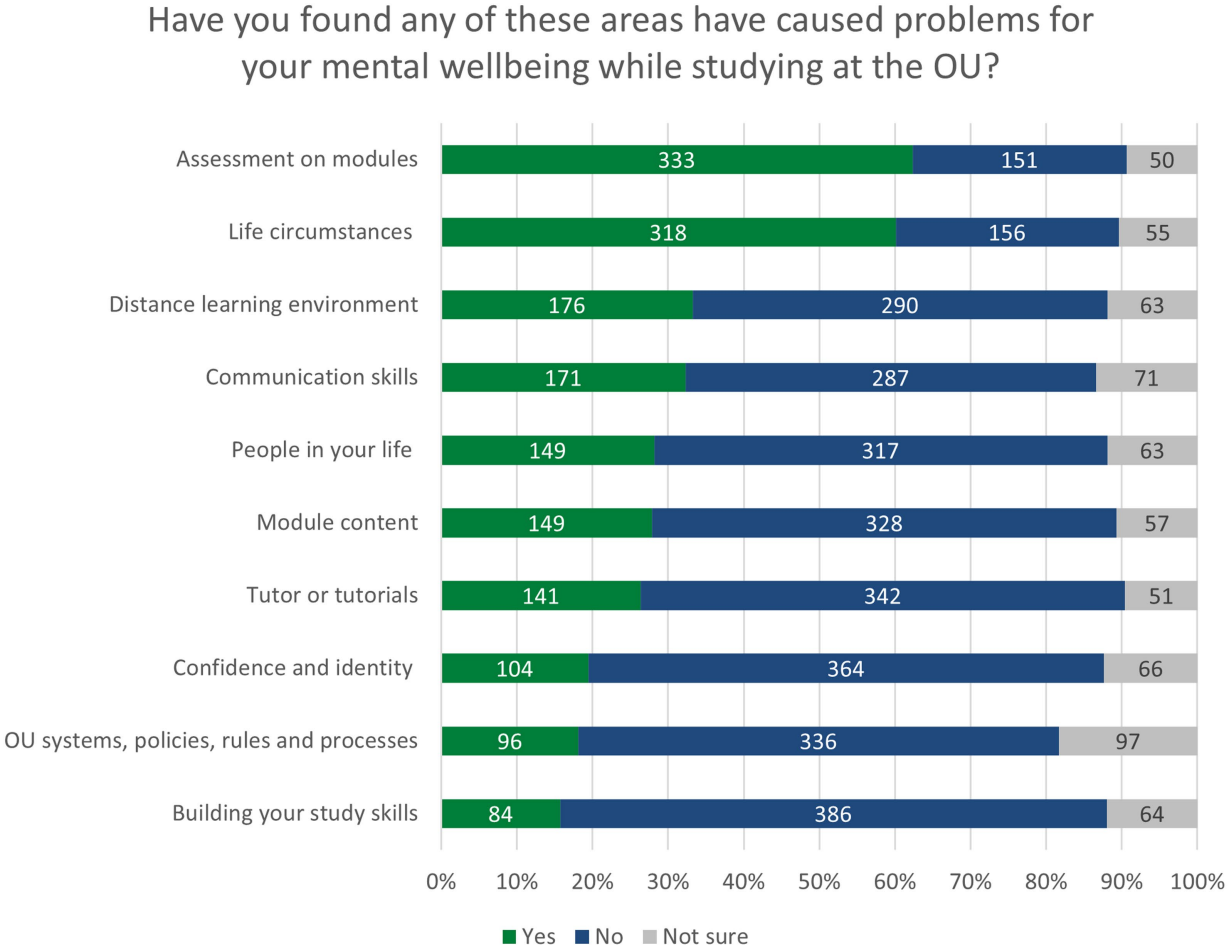
Taxonomy of barriers and enablers to mental wellbeing in distance learning (Lister et al., 2021).

In a small qualitative study, Lister et al. interviewed 16 students who had disclosed their mental health condition about their experiences of studying at a distance learning institution (Lister et al., 2021). Lister et al. mapped barriers and enablers to different aspects of students’ higher education experiences, drawing on the ‘capabilities approach’ (Nussbaum, 2000). The capabilities approach recognises the relationships between wellbeing and external capabilities, such as culture and the impact of affordances or obstacles presented by a person’s environment and context (Nussbaum, 2000; Robeyns, 2005). Later interpretations of the capabilities approach also recognise the role of internal capabilities, such as skills building, and identifying ways skills can be formed, developed, used and measured, in order to contribute to broader capability (Heckman and Corbin, 2016). In a higher education context, external capabilities may relate to environment (such as systems, spaces and people) and study-related capabilities (such as curriculum, assessment and pedagogy), which Lister et al. depicted as a taxonomy, shown in Figure 1. This taxonomy illustrates relationships between barriers and diametrically corresponding enablers, and indicates relationships between adjacent themes within both barriers and enablers.

In the study reported in this paper we seek to build on Lister et al’s study by examining the barriers and enablers experienced by a larger sample of distance education students. The study took place in the Open University (OU), a large UK distance learning university with over 140,000 students. At the time of the study (2020–21), 18,498 (12.4% of) OU students disclosed a mental health condition, and recent reporting showed consistent gaps in attainment (1.3 percentage points) and module completion (15.7 percentage points) for students with mental health difficulties (The Open University, 2019). The survey was part of a larger project to identify changes that could be made to better support students’ mental wellbeing in distance learning environments, study and skills-building; this larger project included staff and student focus groups (Lister, 2021), pilot projects and a staff survey (publication to follow).

## 3. Materials and methods

The project of which this study is part aligns to the critical (or transformative) educational research paradigm, as it seeks not only to understand phenomena but identify ways to redress inequalities inherent within them (Cohen, 2007; Mertens, 2007). It adopts critical pedagogy (Freire, 1970) and the social model of disability (Oliver, 1983) as theoretical frameworks, identifying systemic oppression in educational practice that impacts on student mental wellbeing, and positioning these as barriers to equity that educators have a responsibility to address. In line with this, it holds to the ideology, principles and methods of participatory inquiry (Heron and Reason, 2016), recognising students’ lived experience as expertise.

This paper explores on aspect of the wider project; a survey that aimed to gather data from students on barriers and enablers they had experienced in distance learning, and seek their ideas for changes that could be made. The study and survey instrument were approved by the OU Human Research Ethics committee, Student Research Project Panel, and Data Protection team. The survey instrument is available as supplementary material.

The survey instrument was collaboratively designed with three students who had disclosed mental health conditions, following a participatory design approach. Students worked from a first draft of the instrument, and suggested wording for questions, multiple choice options and refined the question order. An iterative approach was followed, with students collaborating on four drafts before the survey instrument was finalised.

As part of the ethical design, the survey wording and question order were designed to avoid causing distress to participants. The survey opened and closed with general, light-touch questions, aiming to provide a positive onboarding and offboarding experience (e.g., ‘Has your mental health had an effect on your OU studies at all?’ and ‘On the whole, do you find OU study to be good or bad for your mental wellbeing?’) After the onboarding questions, section two of the survey focused on positive effects that study had had on mental wellbeing, encouraging the critical consciousness about the positive role study could play on mental health (i.e., ‘Have you found any of these areas have helped your mental wellbeing while studying at the OU?’ followed by a list of aspects of study with yes/no/not sure options, and a free text question.) The opening text for section two advised students that section three would be asking about negative impacts of study on mental wellbeing, so students were forewarned this would be coming (this included the question ‘Have you found any of these areas have caused problems for your mental wellbeing while studying at the OU’, followed by a list of aspects of study with yes/no/not sure options, and free text question.) Immediately after this, section four focused on support and guidance that was available to students, encouraging them to reflect on what had had a positive impact on them and aiming to raise awareness of any support of which students may not have been aware. The text introducing this section advised that links to all the guidance and support could be found at the end of the survey, aiming to provide practical support to students, while balancing this against the risk of distracting students from completing the survey. The following section asked students’ opinions of specific OU wellbeing initiatives, and the final section asked about broader impacts of distance learning on mental wellbeing, (e.g., ‘In general, how well do you feel your mental wellbeing has been supported by OU module curricula, assessment and the learning activities you take part in?’) aiming to provide distraction and a sense of perspective for any students who may have found it distressing to reflect on negative mental health experiences.

Care was taken to support students; they were advised in the open comment sections that the anonymous nature of the survey meant staff would not be able to respond to any queries raised in the open comment questions, but they were given links to mental health support pages in the question text in case they needed assistance. The survey closed by thanking the students, providing links to the different support options mentioned previously, and wishing students positive mental health and success in their studies.

The survey was piloted with 12 students (eight female, four male) in order to check the validity, particularly in terms of checking that the language was understandable, the questions were framed correctly and that there were no omissions in questions or multiple choice options (Johanson and Brooks, 2010).

### 3.1. Participants

This study sought to gain insight from students both with and without disclosed mental health conditions, using a stratified, random sampling technique. Two stratified random samples were obtained from the University Surveys team; these comprised a total of 5,000 students studying during academic years 2019/20 and 2020/21. The first sample consisted of 2,500 students who had disclosed a mental health condition to the university; the second sample consisted of 2,500 students who had not disclosed any mental health conditions. The samples were stratified to be representative of the broader cohort in terms of gender, ethnicity, faculty and geographic location, with under 1.4% variance.

In total, 584 students responded to the survey, a response rate of 11.68%. The response rate was higher for students disclosing a mental health difficulty; of the 2,500 students who were invited, 340 responded, resulting in a 13.6% response rate from this group compared to a 9.76% (N = 244) response rate from the 2,500 students who did not disclose a mental health condition.

Participant demographics are shown in Table 1, below. Due to small numbers, some of the classifications were later grouped for analysis (i.e., in ‘previous educational qualification,’ the ‘no formal qualification’ group was combined with ‘less than A-levels.’) Socio-economic status was measured using the UK index of multiple deprivation (IMD) which classifies participants’ relative deprivation according to postcode area.

**Table 1:** Survey respondent demographics.

Participant characteristic		Count	%
Mental health disclosure	No	244	42%
	Yes	340	58%
Age	Under 25	120	21%
	26–35	155	27%
	36–45	115	20%
	46–55	121	21%
	56 and over	73	13%
Previous educational qualification	No formal qualifications	14	2%
	Less than A Levels	163	28%
	A Levels or equivalent	142	24%
	HE Qualification	135	23%
	PG Qualification	35	6%
	Not known	95	16%
Gender	Female	432	74%
	Male	152	26%
Ethnicity	Asian	13	2%
	Black	15	3%
	Mixed	20	3%
	Other	9	2%
	Refused	7	1%
	Unknown	9	2%
	White	511	88%
Socio-economic status (IMD, by postcode)	0–20%	121	21%
	20–40%	116	20%
	40–60%	106	18%
	60–80%	100	17%
	80–100%	103	18%
	Non-UK or unknown	38	6%
Disability (other than mental health)	No	500	86%
	Yes	84	14%

### 3.2. Analysis

The survey analysis followed a participatory approach, with students forming part of the analysis team and leading on aspects of the analysis. The survey captured frequency data and open comments, and was analysed using SPSS and NVivo. Frequency data was analysed using descriptive statistics to identify barriers, enablers and impacts. Crosstab analysis of frequency data was used to contrast the findings from different demographic groups, between students disclosing and not disclosing a mental health condition, and students at different stages of study. Pearson’s Chi squared was used to determine statistical significance, with an alpha level of 0.05 for all statistical tests. Open comments in the survey were analysed in NVivo using Thematic Analysis (Braun and Clarke, 2006).

## 4. Results

This section reports the findings of the survey. First, it reports impacts of students’ mental health on their studies, followed by barriers and enablers to mental wellbeing they experienced and the impact that distance learning had on them. Finally, it reports their suggestions for, and prioritisation of, changes to make distance learning more conducive to mental wellbeing.

### 4.1. Mental health in distance learning

**Figure 2:**
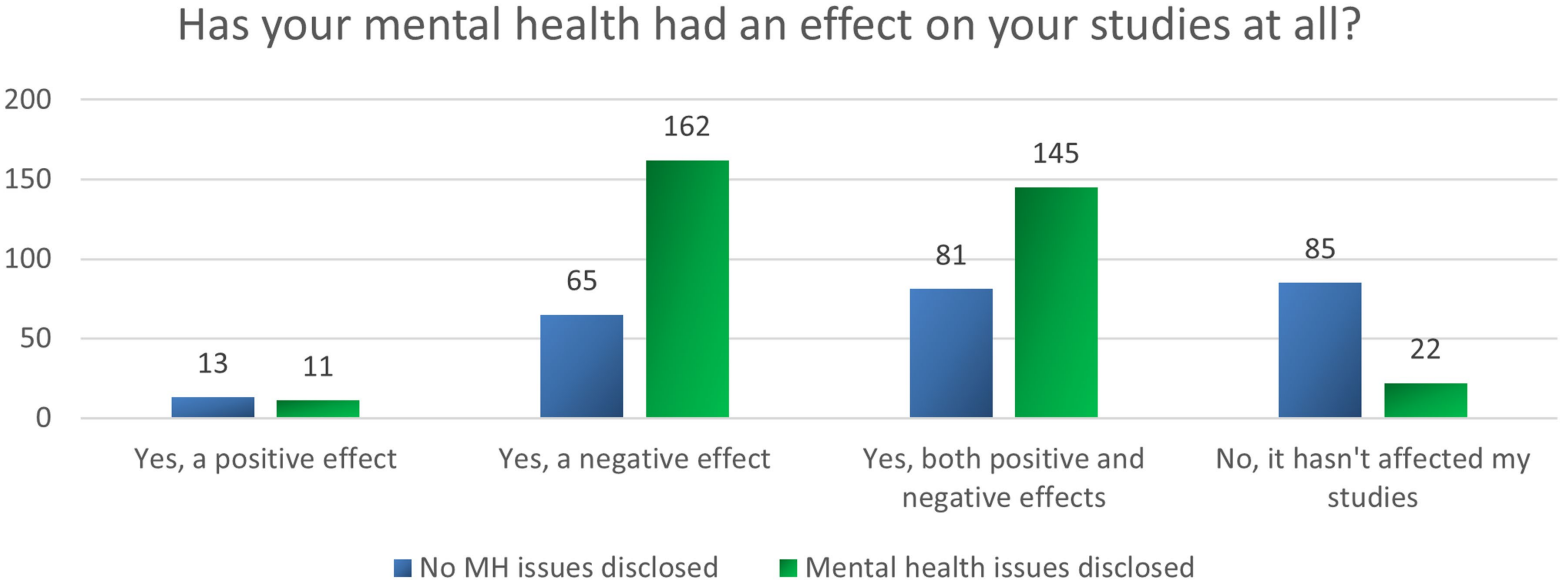
Impact of mental health on studies.

As shown in Figure 2, 87.7% (*N* = 477) of students stated that their mental health had had an impact on their studies; only 4.1% (*N* = 24) reported a positive effect, while 38.9% (*N* = 227) reported a negative effect and 38.7% (*N* = 226) reported that their mental health had had both positive and negative effects on their studies. Students disclosing a mental condition reported significantly more negative and mixed effects, while fewer reported positive or no effects on their studies [*X*^2^ (3, *N* = 584) = 83.304, *p* < 0.001].

### 4.2. Barriers

**Figure 3:**
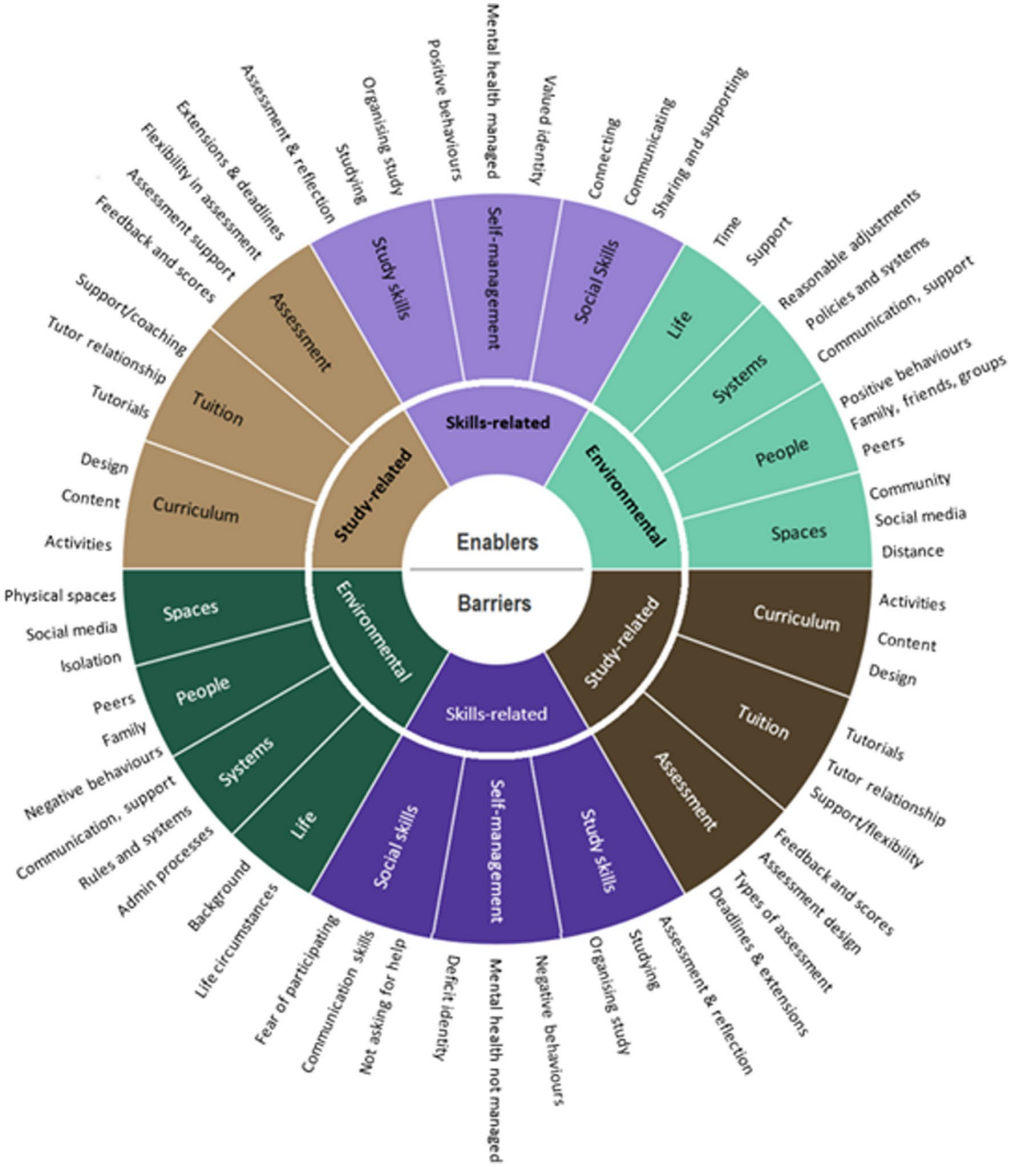
Taxonomy of barriers and enablers to mental wellbeing in study. Figure copyright: Kate Lister, Jane Seale & Chris Douce (CC-BY 4.0).

In the small-scale qualitative study reported by Lister et al. (2021), 10 barriers to mental wellbeing were identified relating to Study, Skills and Environment (see Figure 1). The results from our survey indicate that respondents experienced the same barriers. Certain barriers were experienced by higher numbers of students; 62.4% (*N* = 333) of students found ‘assessment, deadlines or feedback’ had caused problems for their mental wellbeing, and 60.1% (*N* = 318) of students stated that their life circumstances while studying had been a barrier for them. In contrast to this, only 18.1% (*N* = 96) of students found that ‘OU systems, policies, rules and processes; had been a barrier for them, and only 15.7% (*N* = 84) found that building their study skills had been a barrier. The results across all 10 areas are shown in Figure 3.

The responses were analysed for statistically significant variations according to the following criteria:

- Socio-economic status
- Gender
- Ethnicity
- Age
- Mental health disclosure
- Other disability (excluding a mental health disclosure)
- Previous educational qualifications

There were statistically significant differences in nine of 10 areas for students who disclosed a mental health condition. In every area except ‘the distance learning environment,’ students with a disclosed a mental health condition were more likely to experience a barrier than students without a mental health disclosure. These are shown in Table 2, below.

**Table 2:** Survey findings: barriers by mental health declaration.

Category	% barrier all students	% barrier, with MH declaration	% barrier without MH declaration	Pearson’s Chi Square
Assessment on modules	62.40%	71.90%	48.90%	*X^2^* (2, *N* = 534) = 36.452, *p* < 0.001
Your life circumstances in general while you have been studying	60.10%	67.90%	49.30%	*X^2^* (2, *N* = 529) = 28.945, *p* < 0.001
The distance learning environment, forums or student social media	33.30%	35.10%	30.80%	Not significant: *X^2^* (2, *N* = 529) = 1.205, *p* = 0.547
Building skills in communicating with your tutor or your peers	32.30%	40.60%	20.80%	*X^2^* (2, *N* = 529) = 34.652, *p* < 0.001
Module content	28.20%	33.10%	21.30%	*X^2^* (2, *N* = 529) = 15.053, *p* = 0.001
The people in your life while you have been studying	27.90%	31.60%	22.60%	*X^2^* (2, *N* = 534) = 18.1, *p* < 0.001
Tutor or tutorials	26.40%	31.00%	19.90%	*X^2^* (2, *N* = 534) = 19.362*, p* < 0.001
Confidence and identity as an OU student	19.50%	23.00%	14.50%	*X^2^* (2, *N* = 534) = 13.393, *p* = 0.001
OU systems, policies, rules and processes	18.10%	22.70%	11.80%	*X^2^* (2, *N* = 529) = 14.529, *p* = 0.001
Building your study skills	15.70%	17.60%	13.10%	*X^2^* (2, *N* = 534) = 11.535, *p* = 0.003

Four of the barriers were also statistically significant for certain students depending on their age, gender, socio-economic status or whether they disclosed a disability.

‘Building communication skills’ was more likely to be a barrier for:

- Women: 34.4% of women compared to 26.1% of men recorded this as a barrier [*X^2^* (2, *N* = 529) = 8.573, *p* = 0.014].
- Students with low socio-economic status: 41.7% of low SES students recorded this as a barrier, compared to 33.2% of mid-level SES and 25.8% of high SES [*X^2^* (6, *N* = 529) = 15.350, *p* = 0.018].
- Students between 26 and 45 years old: 37.9% recorded this as a barrier, compared to 34.3% of students under 25 and 23.9% of students over 46 [*X^2^* (4, *N* = 529) = 9.623, *p* = 0.047].

‘Assessment’ was more likely to be a barrier for:

- Younger students: 77.9% of students under 25 years old recorded this as a barrier, compared to 68.9% of students 26–45 years old and 45.2% of students over 46 [*X^2^* (4, *N* = 534) = 39.224, *p* < 0.001].
- Students with a disability other than mental health: 60.1% recorded this as a barrier, compared to 49.9% who disclosed no disability or only mental health [*X^2^* (2, *N* = 534) = 6.405, *p* = 0.041].

‘Module content’ was more likely to be a barrier for students disclosing a disability other than mental health, with 37.5% (*N* = 30) stating it was a barrier, compared to 26.2% (*N* = 119) without a disability or disclosing mental health issues alone [*X^2^* (2, *N* = 534) = 7.726, *p* = 0.021].

‘Life circumstances’ were more likely to be a barrier for younger students, with 74.5% (*N* = 76) of students under 25 recording this as a barrier, compared 62.6% of students 26–45 (*N* = 152) and 48.9% (*N* = 90) of students over 46 [*X^2^* (4, *N* = 529) = 22.393, *p* < 0.001].

Students also provided free text responses about ‘anything that had a negative impact on your mental health while studying at the OU.’ This resulted in 301 comments, which were analysed in NVivo and led to 427 coded references. Numbers and examples of coded references per theme are shown in Table 3.

**Table 3:** Open comments referring to barriers.

Barrier category	Theme	Coded references	Example
Environment	Negative life circumstances	139	‘COVID and having to shield, not having seen my family outside of my partner since February. It’s all felt very isolated’, ‘Emotional abuse, getting kicked out, having to work so many hours’, ‘My ‘day job’ workload; my younger sister was diagnosed with terminal cancer, and the general impact of coronavirus-having to look at redundancies for staff etc.’
	OU systems	15	‘not receiving my textbooks weeks after my course has started’, ‘long delay in hearing about the exam arrangements’
	People	36	‘My family at home have caused problems with my mental health as they have been critical and unsupportive.’
	Spaces, isolation	26	‘It can be very lonely and isolating when distance learning.’
Skills	Confidence and identity	11	‘Comparing my performance to other students.’
	Social skills	6	‘I find it almost impossible to engage with my peers through the forums or participate during tutorials. Even though I might have ideas and be able to contribute to a discussion I cannot bring myself to draw any attention to myself. It’s hard to just email my tutor if I’m struggling. This is nothing to do with them, or any aspect of the OU, it’s just my anxiety is appalling.’
	Study skills	13	‘I found using the computer system for submitting work stressful at times. This due to my lack of knowledge regarding computers’
Study	Assessment, deadlines, feedback	70	‘Pressure of exams’, ‘The fear of failing my assignments’, ‘the essay questions have been very vague with vague guidance and it has caused a lot of stress trying to work out exactly what is required’, ‘Find assessments very stressful’, ‘deadlines had negative impact’, ‘struggle with reading assessment feedback, good or bad can be triggering’
	Curriculum	59	‘I find that I’m not able to keep up the pace with the suggested deadlines of topics in the modules and that causes me a great deal of distress.’
	Tuition	52	‘My last tutor for my final year was quite absent and not particularly supportive when I struggled with my project.’

### 4.3. Enablers

**Figure 4:**
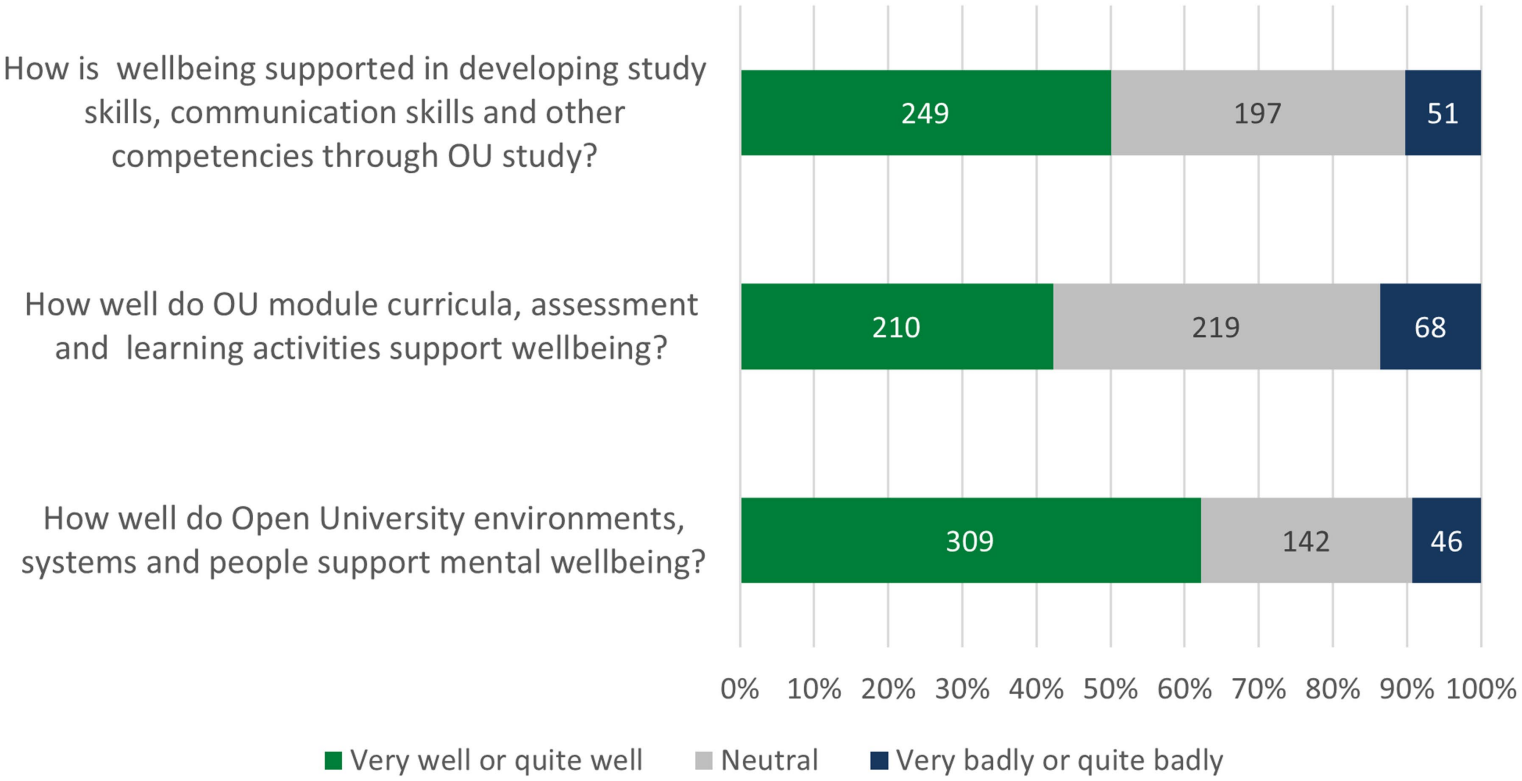
Impact of study on wellbeing.

In the small-scale qualitative study reported by Lister et al. (2021), 10 enablers to mental wellbeing were identified, relating to Study, Skills and Environment (see Figure 1). The results from our larger survey indicate that respondents experienced the same enablers. The numbers and percentages of students reporting positive impacts or enablers were generally higher than those reporting barriers. As with barriers, certain enablers were experienced by higher numbers of students; 63.7% (*N* = 358) of students found that building their study skills had supported their mental wellbeing, and 64.8% (*N* = 355) of students stated that the people in their lives has been a positive factor. In contrast to this, only 34.9% (*N* = 191) of students found that life circumstances had been an enabler for them, and only 24.6% (*N* = 135) found that ‘OU systems, policies, rules and processes’ had played a positive role. The results across all 10 areas are shown in Figure 4.

An interesting finding, in stark contrast to the barriers, was that having disclosed a mental health declaration did not make a statistically significant difference to students’ experience of enablers. The only area where students with mental health responded significantly differently was in assessment, as students disclosing a mental health condition were less likely to experience assessment as an enabler: 48.9% (*N* = 160) of students with mental health conditions declared it was not an enabler, compared to 39.6% (*N* = 93) of students without a disclosure [*X*^2^ (2, *N* = 562) = 10.857, *p* = 0.004]. This implies that enablers are positively experienced in general by students, while barriers appear more keenly felt by students with diagnosed mental health difficulties.

In contrast to this, participants’ age, socio-economic status, gender, and ethnicity had a more significant impact on the responses than a mental health disclosure. Age was significant in five of the 10 areas, with younger students less likely to experience enablers (shown in Table 4.)

**Table 4:** Survey findings: enablers by age.

Category	Overall %, all students	Under 25	26–45	Over 46	Chi^2^
Assessment on modules	37.19%	27.20%	35.80%	45.20%	*X^2^* (4, *N* = 562) = 11.740, *p* = 0.019
Life circumstances	34.85%	26.60%	32.30%	43.10%	*X^2^* (4, *N* = 548) = 11.173, *p* = 0.016
Communication skills	40.15%	38.50%	34.30%	48.90%	*X^2^* (4, *N* = 548) = 10.703, *p* = 0.030
Module content	62.10%	52.60%	58.50%	72.90%	*X*^2^ (4, *N* = 562) = 20.043, *p* < 0.001
Tutor or tutorials	57.30%	50.00%	53.10%	67.60%	*X^2^* (4, *N* = 562) = 15.992, *p* = 0.003

Socio-economic status was a significant factor in two areas, with students with lower socio-economic status (i.e., in the bottom 20% of the IMD) less likely to experience enablers in:

- module content: 53.4% of low SES students rated this as an enabler, compared to 64.0% mid-SES and 62.0% high SES [*X*^2^ (6, *N* = 562) = 13.787, *p* = 0.032].
- life circumstances: 28.1% of low SES students rated this as an enabler, compared to 32.0% mid-SES and 45.9% high SES [*X*^2^ (6, *N* = 548) = 16.402, *p* = 0.012].

Gender was a significant factor in one area, building study skills. 65% of women found building and developing their study skills an enabler compared to 59.9% of men, and 27.5% of men said it was not an enabler for them compared to 16.2% of women [*X*^2^ (2, *N* = 562) = 9.838, *p* = 0.007].

Ethnicity was significant in one area: assessment. Black and minority ethnic students were less likely to find assessment an enabler with only 30.4% stating this compared to 38.3% of white students [*X*^2^ (4, *N* = 562) = 11.947, *p* = 0.018].

Students also provided free text about ‘anything that helped your mental health while studying at the OU.’ The 321 comments received were analysed in NVivo, resulting in 384 coded references that broadly corresponded with the taxonomy and the 10 themes in the survey. The numbers and examples of coded references per theme are shown in Table 5.

**Table 5:** Open comment references to enablers.

Enabler category	Theme	Coded references	Example
Environment	Negative life circumstances	1	‘Being retired!’
	OU systems, comms and support	33	‘Big white wall’, ‘The student support team. Always available and very understanding’, ‘DSA mentor’
	People	49	‘I feel that my dad and stepmum and mum have been very proud of me which has boosted my self confidence’
	Spaces, distance	30	‘A familiar environment while studying has helped’
Skills	Confidence and identity	84	‘It makes me feel like my life is “going somewhere”’, ‘the status of being a postgraduate student instead of a crazy benefit claimant’, ‘I feel proud that I’m studying and getting good marks. I’m possibly a lot smarter than I think I am. I’ve more confidence in my own abilities.’
	Study skills	5	‘Building skills and giving me something to do at home’
Study	Assessment, deadlines, feedback	32	‘The deadlines to ensure I have been keeping on track’, ‘being reassured with my high marks, helped my anxiety’
	Curriculum	75	‘Just the study is a distraction from other problems in my life.’ ‘Learning new material was exciting. I found the readings and activities very interesting.’
	Tuition	75	‘My tutor has been brilliant and the tutorials have helped immensely’

### 4.4. Impact of study on wellbeing

Overall, 71% of students (*N =* 353) stated that study had an overall positive impact on their mental health, implying that the impact of enablers outweighed that of the barriers. This was felt across all demographics; the only one showing slight significant difference was age [*X*^2^ (4, *N* = 497) = 10.530, *p* = 0.032]. Students in the ‘under 25’ and ‘26–45’ age brackets were slightly more likely to say that study was neutral or bad for their mental wellbeing, but were still in the minority; 64.2% (*N* = 61) of students under 25 and 67.2% (*N* = 154) of students between 26 and 45 states OU study was good for their mental wellbeing.

This was similar when looking at specific groups of barriers and enablers. For example, when asked about skills-related barriers/enablers, 50.1% (*N* = 249) of students felt that their mental wellbeing had been well or very well supported as they developed study skills, communication skills and other competencies through study, and 39.6% (*N* = 197) were neutral. There were no significant differences within demographics.

In relation to study-related barriers/enablers, 42% (*N* = 210) of students stated their wellbeing had been well or very well supported by module curricula, assessment and the learning activities, and 44.1% (*N* = 219) were neutral. However, students disclosing a mental health condition were slightly less likely to state this, with 41.3% (*N =* 121) saying ‘well’ or ‘very well’ and also 41.3% (*N* = 121) being neutral [*X*^2^ (2, *N* = 497) = 8.631, *p* = 0.013]. This may relate to the strong differences around experiences of assessment for students with mental health issues.

Finally, in relation to environmental barriers/enablers, 62.2% (*N* = 309) of students stated their wellbeing had been well or very well supported by OU environments, systems and people; 28.6% (*N* = 142) saying they were neutral and 9.3% (*N* = 46) saying it has been badly supported in this area. Interestingly, students disclosing a mental health condition were more polarised in this area, with 67.2% (*N* = 182) saying ‘well’ or ‘very well’ and 11.6% (*N* = 34) saying ‘badly’ or ‘very badly’ [*X*^2^ (2, *N* = 497) = 20.918, *p* < 0.001].

### 4.5. Suggestions for changes

Students were asked an open question about ‘things you would like the OU to do to support students’ mental wellbeing in studying.’ 162 students (28%) gave a response to this question. These were coded in NVivo and clustered into themes, using Braun and Clarke’s Thematic Analysis as a methodology (Braun and Clarke, 2006). These free text responses resulted in 264 references coded to 77 different codes, clustered into 12 themes under five overarching categories:

- Study-related changes (N = 87)
- Environmental changes (N = 95)
- Changes or improvements to support (N = 61)
- Skills-related changes (N = 1)
- No changes suggested (N = 20).

**Figure 5:**
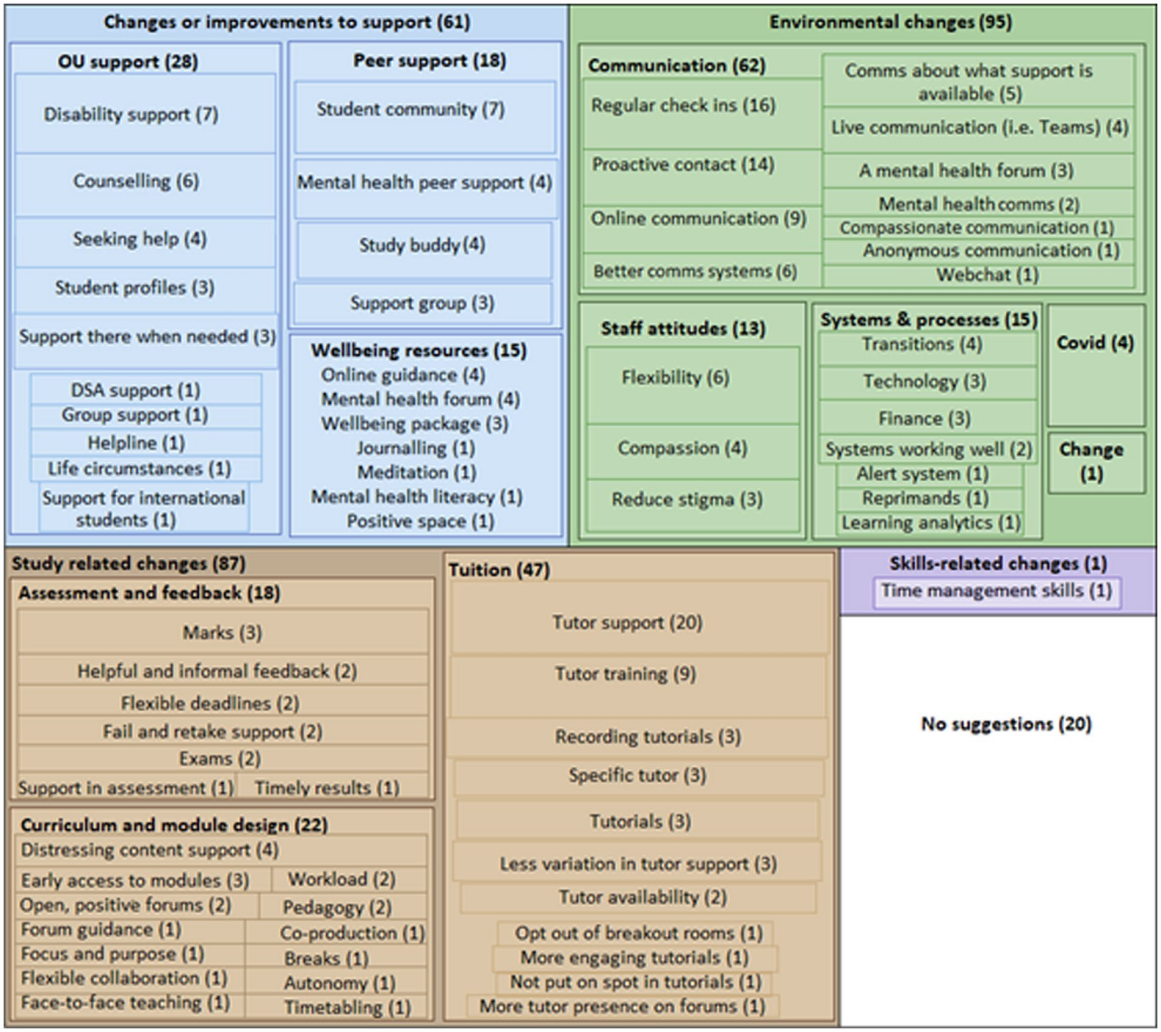
Suggestions for changes to university environments, study support and study practices to enhance student wellbeing.

Figure 5 shows a visualisation of themes within these categories, and selected examples are explored in more detail in the following section.

‘Study-related changes’ contained sub-themes relating to assessment and feedback, curriculum, and tuition and tutor support. The most populated theme was ‘tuition’, with 47 coded references. These included suggestions to improve tutor support, such as:

*‘Have a unified approach from tutors. In my last module I had a fantastic, supportive tutor but some of my peers had tutors who were very unhelpful and it caused them a lot of stress’*

Suggestions for changes to curriculum (N = 22) included supporting students to manage distressing content, e.g.:

*‘Offering trigger warning on potentially upsetting videos or resources.’*

*‘The occasional contact by the tutor, ideally by phone, to the student, may pre-empt difficulties and the opportunity to discuss topics that may have, unexpectedly, distressed the student.’*

With regard to assessment and feedback (N = 18), students asked for changes to:

- Feedback (i.e., ‘More helpful feedback and more consistent feedback’)
- Flexible deadlines (i.e., ‘Be more flexible with final deadlines and EMAs’)
- Support for failing and retaking (i.e., ‘When I failed an exam I had no support or contact of any kind from tutor or the OU. Better support is needed if we fail an exam.’)
- Exams (i.e., ‘Consider making exams easier to manage for those with mental health issues’).

With regard to environmental changes, a key theme was ‘communication;’ with 62 coded references, this was the most populated theme. Suggestions included regular check ins (e.g., ‘Just check once in a while if students are coping or need more support’) and proactive contact (e.g., ‘Having people to reach out and actively engage with students who are clearly not engaging, not attending, are falling behind or performing poorly so ask them, non-judgementally and without threatening them with expulsion, whether there is any support that they need’).

Another theme under ‘environmental changes’ was systems and processes, with 15 coded references. Suggestions related to transition processes (e.g., ‘Not scaring students when they step up from level 1 to level 2 with a whole list of things which they are supposed to already know - which then later come up in the course. This made me very anxious.’); finance (e.g., ‘I would suggest that they should make more clarification on the Study finances that are available for students.’), and systems working well (e.g., ‘It would be helpful if the tutorial system had stayed the same I e being able to access any tutorials.’) Other themes under ‘environmental changes’ related to staff attitudes (N = 13), COVID-19 (N = 4) and general change (N = 1.)

There were 61 references to ‘changes or improvements to support’. Of these, 28 related to OU support (such as ‘Proactively seek out dyslexic students before their studies begin’); 18 related to peer support (i.e., ‘introduce study buddies to help feel less isolated and connections to others - to improve mental health not for learning/improving study outcomes’) and 15 related to mental health support (such as ‘I support having mental wellbeing resources that students can explore on the website if needed and tutors can refer students there if needed.’)

Finally, there was one ‘skills-related’ suggestion, relating to support developing time management skills.

*‘More guidance on how to organise a study timetable for students with jobs that are not a standard 9am-5pm - i.e. how to balance it out but not overload oneself, and give examples of this. It took me years to learn this myself through trial and error, so more guidance would have helped (but the guidance is a lot better now than when I first started anyway).’*

### 4.6. Prioritisation of areas for change

Students were asked how well, in general, they felt their wellbeing was currently supported in three overarching areas:

- OU environments, systems and people
- OU module curricula, assessment and learning activities
- Developing study skills, communication skills and other competencies through OU study

**Figure 6:**
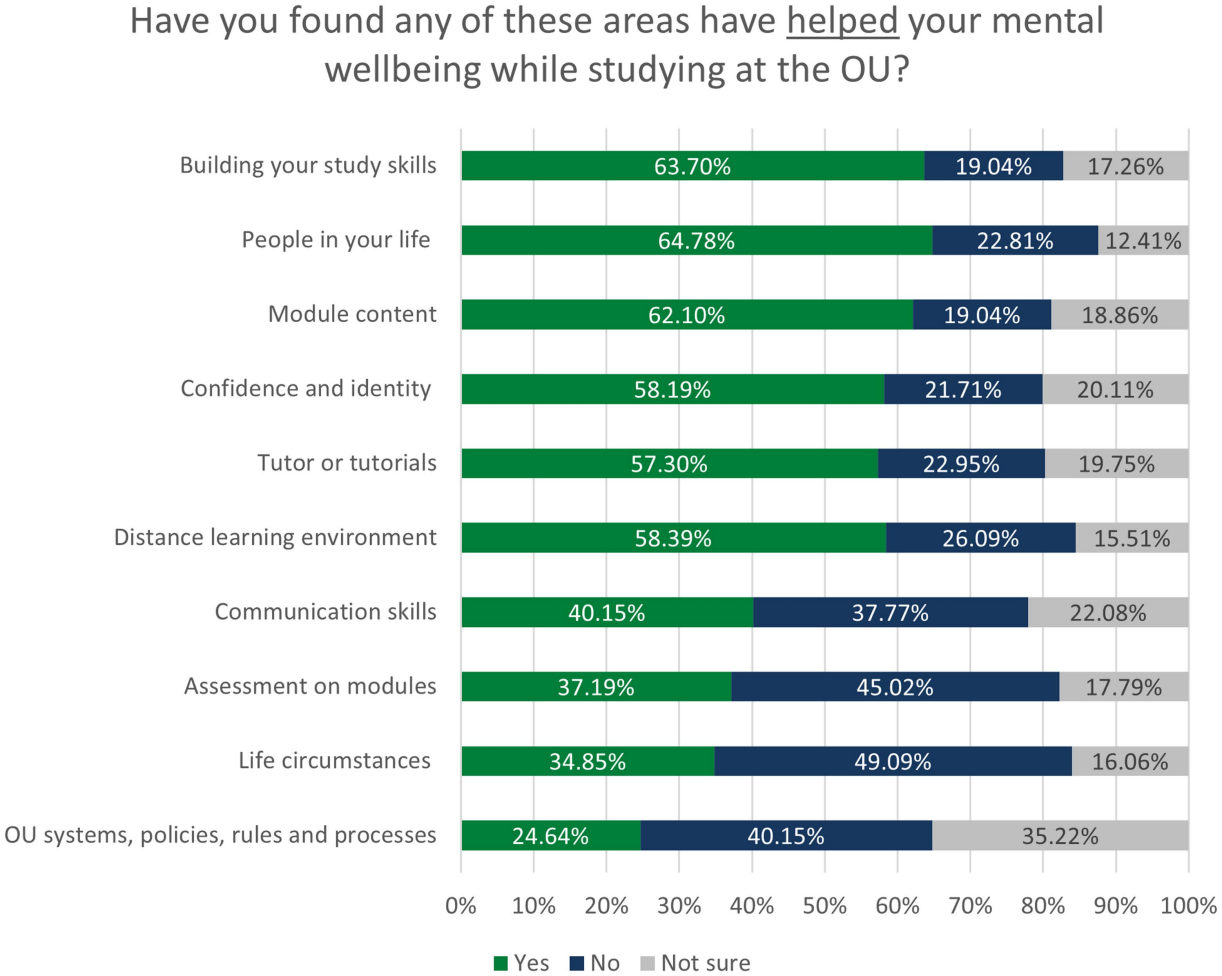
Factors that enhanced student wellbeing in study.

These questions aimed to identify the areas where students felt their wellbeing was less supported, in order to identify priority areas for future solutions. The results are shown in Figure 6.

Very few students felt badly supported. However, the area where students generally felt less well supported was OU module curricula, assessment and learning activities. This implies that action should be prioritised in this area.

## 5. Discussion

This study aimed to answer the following three research questions:

1. What barriers to mental wellbeing do distance learning students experience, and are particular demographic groups more likely to experience these barriers?
2. What enablers to mental wellbeing do distance learning students experience, and are particular demographic groups more likely to experience these enablers?
3. What changes do distance learning students recommend that would enhance mental wellbeing in distance learning?

### 5.1. Barriers and enablers experienced

The survey data supports the taxonomy model proposed by Lister et al. (2021), in which aspects of the higher education experience can be either barriers and enablers, depending on their design and how they are experienced by students. Students stated they experienced barriers and enablers to wellbeing in all the areas of Lister et al’s taxonomy, and aspects of the higher education experience can be either barriers and enablers, depending on their design and how they are experienced by students. The survey adds to this model by providing data on the numbers of students experiencing different barriers and enablers.

Assessment is clearly the most critical barrier for students disclosing mental health difficulties, with 71.90% stating this had been a barrier to their wellbeing. This is particularly significant when considered with the findings that students felt their wellbeing was least well supported in the area of module curricula, assessment and learning activities, and strongly implies that action should be prioritised in this area. The open comments show that assessment design, assessment type, deadlines and feedback can cause stress, anxiety, or contribute to barriers to wellbeing. This broadly supports the literature; many studies recognise that assessments are a trigger point for student stress and anxiety (i.e., Galante et al., 2018; Jones et al., 2018; Hill et al., 2019), and that there is a need for assessment practices in HE to evolve, to be more inclusive and less likely to provoke distress (Boud and Falchikov, 2007; Hanesworth et al., 2019). However, some participants found barrier themes to be enablers; some aspects of assessment were found to support or enable wellbeing. Open comments suggest that deadlines could be helpful, and pride in grades could have a positive impact on student wellbeing.

In contrast to assessment, it was interesting that university systems were not perceived to be a barrier or an enabler for many students, ranking second lowest in terms of barriers and lowest in terms of enablers. The few open comments related to particular problems experienced, or additional support received, suggesting that day-to-day systems may be unperceived by students, and not considered to be a barrier or enabler. This contrasts with the literature, where university systems are often seen to be a barrier (Tinklin et al., 2005; Markoulakis and Kirsh, 2013; Coughlan and Lister, 2018).

Overall, the survey revealed insights into the challenges of distance learning and the impacts on wellbeing. The open comments reveal that Covid-19 exacerbated the isolation and stress students felt as distance learners. However, students also reported positive impacts of distance learning as enablers to wellbeing, particularly around building their study skills, the people in students’ lives and their curriculum and module content. This aligns interestingly with the literature, which has found learning can have a positive impact on wellbeing, particularly with older adults (Field, 2009; Waller et al., 2018).

### 5.2. Demographic groups experiencing barriers and enablers

A clear theme emerging from the demographic analysis of data was that while barriers disproportionately affected students disclosing mental health difficulties (and to a lesser extent other disabilities), enablers were experienced more generally, with no significant difference between students with and without disability and mental health disclosures. This supports the contention often found in disability and inclusion literature that inclusive practice benefits *all* students, not just those with disabilities or particular study needs (Male, 1996; Rose and Meyer, 2002; Boyle, 2011; Fovet, 2018; Haynes, 2019; Lopez-Gavira et al., 2019).

The data implies that barriers in general were disproportionately experienced by minority or disadvantaged groups; in particular, students with a mental health condition, students with low socio-economic status, Black or ethnic minority students, and students with a disability other than mental health. Women and younger students were also disproportionately affected. The findings about women appear to support the general literature, as multiple survey studies have found women more likely to express difficulties with mental wellbeing in study (Bernhardsdóttir and Vilhjálmsson, 2013; Mokhtari et al., 2013; Evans et al., 2018). However, literature about mental health and age in higher education tends to position more mature students as more vulnerable to mental health difficulties (Wong and Kwok, 1997; Swain and Hammond, 2011; Busher and James, 2020), so the finding in this survey that younger students appear more vulnerable was interesting, particularly in the context of lifelong education. It may be that this is a particular feature of the distance learning environment; that distance learning is more challenging for younger students and that this can lead to barriers to wellbeing. It would be interesting to replicate this survey in a face-to-face learning institution, or in other lifelong education contexts, and identify if a similar pattern emerged.

### 5.3. Changes to enhance mental wellbeing in distance learning

This study also aimed to identify the changes distance learning students suggested to enhance mental wellbeing in distance learning. The most populated theme for suggestions for change related to communications, followed by tuition and tutor practice. This broadly supports the study by Baik et al., in which the highest number of student recommendations for change related to ‘Academic teachers and teaching practices,’ while changes to communication was the third most popular recommendation (Baik et al., 2019). However, ‘Assessment’ was of low priority in Baik et al’s study, the second-to-least populated, while it was the fourth most populated of twelve themes in this study.

Another interesting finding from the open question on suggested changes was the lack of focus on changes to students’ skills. There was only one suggestion to improve skills, compared to 87 suggestions for study-related changes, 95 suggestions for environmental changes and 61 suggestions for support-related changes. This contrasts sharply with the literature, much of which focuses on skills building as a way to build resilience and manage mental health (Hewitt and Stubbs, 2017; AMOSSHE, 2018; Barrable et al., 2018; Holdsworth et al., 2018). Referring back to the capabilities approach (Nussbaum, 2000), the skills-based solutions represent building internal capabilities, while the environmental, support and skills-related solutions suggest changes that facilitate external capabilities. It appears that the broader literature in the sector is more likely to perceive solutions require effort from the students in building internal skills and resilience. However, students appear to take the view that solutions should come from staff and the university in terms of changing practice, offering additional support and supporting external capabilities. It may be that both parties are to some extent shifting the burden of change to the other party. This concept should be explored further in a future study.

### 5.4. Limitations

There were limitations to this study. This study sought student voices, meaning the voices of Open University staff were missing, although staff voices were prioritised in an earlier phase of the overall study (Lister, 2021; Lister and McFarlane, 2021). Furthermore, low response numbers were received from certain demographic groups, such as Black or ethnic minority students, meaning their experiences are not adequately represented, their voices not sufficiently heard. Another limitation was (as with any survey) the participants were self-selecting, and this resulted in a volunteer bias where a larger number of students with mental health issues responded to the survey.

## 6. Conclusion

This paper has presented the findings from a survey sent to 5,000 students to identify barriers and enablers to wellbeing and ideas for change. This study challenges individualistic models of student wellbeing by identifying that assessment practices represent significant barriers to student mental health, and advances previous knowledge in this area by identifying enablers to wellbeing in building study skills, and in curriculum and module content. A clear message for educational providers, especially distance learning institutions, is that assessment strategies and practices should be prioritised as an area for action to better support student wellbeing in distance learning.

This study also revealed significant demographic differences in how students experience barriers and enablers and how likely they feel they are to benefit from solutions. Students with mental health difficulties were consistently more likely to experience barriers and more likely to feel they would benefit from solutions. Furthermore, enablers were more likely to be felt consistently by students, regardless of demographics. This sends a clear message to educational providers that prioritising enablers to student wellbeing in study environments and practices may be beneficial for all students, not only those with mental health difficulties or other particular study needs.

This study is one part of a larger project to identify changes that could be made to better support students’ mental wellbeing in distance learning environments, study and skills-building. More work is needed to identify solutions that can be embedded in practice, particularly in the area of assessment, to make distance education and lifelong learning more conducive to student mental wellbeing.

## Data availability statement

The datasets presented in this article are not readily available because this data is confidential and as such is not openly shared. Anonymised data may be available upon request. Requests to access the datasets should be directed to klister@arden.ac.uk.

## Ethics statement

The studies involving human participants were reviewed and approved by The Human Research Ethics Committee, Open University. The patients/participants provided their written informed consent to participate in this study.

## Author contributions

All authors contributed to the survey instrument design and writing this paper. All authors contributed to the article and approved the submitted version.

## Funding

This project was internally funded through the Open University Quality Enhancement and Innovation fund.

## Conflict of interest

The authors declare that the research was conducted in the absence of any commercial or financial relationships that could be construed as a potential conflict of interest.

## Publisher’s note

All claims expressed in this article are solely those of the authors and do not necessarily represent those of their affiliated organizations, or those of the publisher, the editors and the reviewers. Any product that may be evaluated in this article, or claim that may be made by its manufacturer, is not guaranteed or endorsed by the publisher.
